# Study of interaction energies between residues of the active site of Hsp90 and geldanamycin analogues using quantum mechanics/molecular mechanics methods

**DOI:** 10.12688/f1000research.20844.2

**Published:** 2020-04-16

**Authors:** Ricardo Vivas-Reyes, Alejando Morales-Bayuelo, Carlos Gueto, Juan C. Drosos, Johana Márquez Lázaro, Rosa Baldiris, Maicol Ahumedo, Catalina Vivas-Gomez, Dilia Aparicio

**Affiliations:** 1 Grupo de investigación (CIPTEC), Facultad de Ingeniería, Programa de Ingeniería de Procesos, Fundación Universitaria Tecnológico Comfenalco, Cartagena, Bolívar, Colombia; 2 Grupo de Química Cuántica y Teórica, Programa de Química, Facultad de Ciencias Exactas y Naturales, Universidad de Cartagena, Cartagena, Bolívar, Colombia; 3 Grupo GINUMED, Facultad de Salud, Programa de Medicina, Corporación Universitaria Rafael Núñez, Cartagena, Bolívar, Colombia; 4 Grupo de Bioinorganica, Programa de Química, Facultad de Ciencias Exactas y Naturales, Universidad de Cartagena, Cartagena, Bolívar, Colombia; 5 Grupo de Microbiología Clínica y Ambiental, Facultad de Ciencias Naturales y Exactas, Programa de Biologia, Universidad de Cartagena, Cartagena, Bolívar, Colombia

**Keywords:** Hsp90, geldanamycin analogues, PM6, 3D-QSAR, QM/MM approach, Molecular Quantum Similarity.

## Abstract

**Background: **Heat shock protein (Hsp90KDa) is a molecular chaperone involved in the process of cellular oncogenesis, hence its importance as a therapeutic target. Geldanamycin is an inhibitor of Hsp90 chaperone activity, which binds to the ATP binding site in the N-terminal domain of Hsp90. However, geldanamycin has shown hepatotoxic damage in clinical trials; for this reason, its use is not recommended. Taking advantage that geldanamycin binds successfully to Hsp90, many efforts have focused on the search for similar analogues, which have the same or better biological response and reduce the side effects of its predecessor; 17-AAG and 17-DMAG are examples of these analogues.

**Methods: **In order to know the chemical factors influencing the growth or decay of the biological activity of geldanamycin analogues, different computational techniques such as docking, 3DQSAR and quantum similarity were used.  Moreover, the study quantified the interaction energy between amino acids residues of active side and geldanamycin analogues, through hybrid methodology (Autodock-PM6) and DFT indexes.

**Results: **The evaluation of interaction energies showed that the interaction with Lys58 residue is essential for the union of the analogues to the active site of Hsp90, and improves its biological activity. This union is formed through a substituent on C-11 of the geldanamycin macrocycle. A small and attractor group was found as the main steric and electrostatic characteristic that substituents on C11 need in order to interact with Lys 58; behavior was observed with hydroxy and methoxy series of geldanamycin analogues, under study.

**Conclusion:** This study contributes with new hybrid methodology (Autodock-PM6) for the generation of 3DQSAR models, which to consider the interactions between compounds and amino acids residues of Hsp90´s active site in the alignment generation. Additionally, quantum similarity and reactivity indices calculations using DFT were performed to know the non-covalent stabilization in the active site of these compounds.

## Introduction

In recent years, molecular chaperones have been of great interest to the scientific community, since these compounds play an important role in apoptosis and cellular oncogenesis. In addition, they maintain the correct folding and three-dimensional conformation of proteins in the cell and control the balance between the synthesis and degradation of many proteins ^1^. Such chaperones include the heat shock protein of 90KDa (Hsp90), which is ATP dependent ^2– 6^. Hsp90 are chaperones that are highly conserved in many species ^7– 9^. Under normal conditions in the cell, Hsp90 are present between 1–2% and can be found in the cytosol, nucleoplasm, endoplasmic reticulum and mitochondria ^10, 11^. Moreover, these chaperones are involved in the maturation of oncogenes and play an important role in the survival, invasion, proliferation, metastasis and angiogenesis of cancer cells ^12^. The expression of Hsp90 is associated with many types of tumors, including breast cancer, pancreatic carcinoma, leukemia, systemic lupus, as well as resistance to many drugs ^13^. The viability of Hsp90 as a therapeutic target in cancer is defined by: (1) its participation in folding and stabilization of a wide range of proteins involved in oncogenesis and malignant progression, which is important for maintenance of cancer cells; (2) the micro-environmental conditions found in tumor hypoxia such as low pH and a bad nutritional status, which tend to destabilize proteins, being necessary for chaperone activity; and (3) an increase in Hsp90 concentration in cancer cells ^14^

Structurally, Hsp90 have three domains, which are important for its chaperone activity, which are: (1) the N-terminal domain, highly conserved (25 KDa) whose function is ATP union; (2) the middle domain, which is the site of union of “client proteins”; and (3) the c-terminal domain, responsible for the homodimerization of theHsp90 ^15^. Thus, Hsp90 chaperone activity can be inhibited when some of these sites are blocked. In fact, Hsp90 inhibitors have been categorized according to their mode of action: (1) blocking of the binding site of ATP, (2) breakdown of the interactions of the co-chaperone/Hsp90, (3) antagonism of the union between the client protein/Hsp90, and (4) interference with the modifications post-transactional of Hsp90 ^14^. In this way, Hsp90 chaperone activity inhibition is a promising target for development of anticancer drugs ^13, 16^. One of these inhibitors is geldanamycin, an antitumor antibiotic that has the ability to join the binding site of ATP in the N-terminal of the Hsp90 domain, triggering the loss of chaperone Hsp90 ^7, 14, 17^. However, geldanamycin shows a high cytotoxicity testing power, as well as hepatotoxicity in clinical trials, hence its disuse ^8, 14, 18^. Recently, some analogues of geldanamycin have been synthesized, which showed less toxic side effects and better anticancer activity. Among these analogues, we found: 17-allylamino-17-demethoxygeldanamicyn (17AAG) and 17-desmethoxy-17-N, N dimethyl amino ethyl amino geldanamycin (17DMAG) ^19, 20^. Thus, this research focused on the search of chemical parameters that can help design the best geldanamycin analogues, which could make the toxic effects of its predecessor disappear. This paper presents an analysis based on a combination of different techniques and computational methods (docking, hybrid methodology and, 3DQSAR s) and quantum similarity, with the purpose of shedding some light on the role of the changes affecting the activity of geldanamycin analogues when modifying substituents of C-11 and C-17 of the macrocycle position.

## Methods

### Molecular mechanics approach

**Figure 1.**
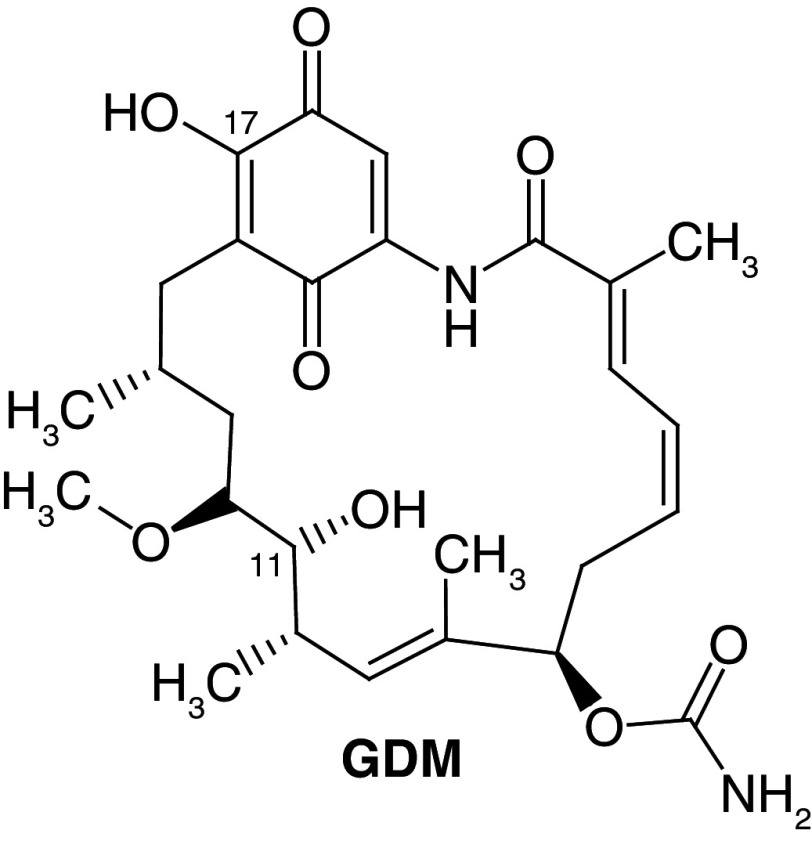
Structure of geldanamycin. The carbon atom C-11 and C-17s of the macrocycle of the geldanamycin are numerically indicated.

***Study setting***. Experimental activity data was taken from the work of Tian *et al.* ^21^. In this paper, the synthesis of 48 geldanamycin analogues was reported. However, only 43 analogues were considered, because only these analogues had IC _50_ values reported. In Figure 1, geldanamycin’s structure as well as the position of carbon atoms C-11 and C-17s are shown. These positions are responsible for the difference between analogues. In Table 1, the substituents of C11 and C17 of each analogue are tabulated with its respective biological activity expressed as pIC50.

**Table 1.** Substituents of C-11 and C-17s positions of geldanamycin analogues.

Analogues	Substituent C11	Substituent C-17	pIC _50_
**GDM**	OH	OMe	7.39
**1a**	OH	NH _2_	7.48
**1b**	OH	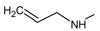	7.48
**1c**	OH	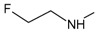	7.77
**1d**	OH	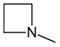	7.62
**1e**	OH	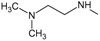	7.62
**1f**	OH	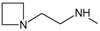	7.59
**1g**	OH	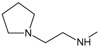	7.15
**1h**	OH	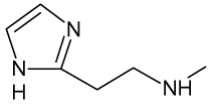	7.01
**2**	OMe	OMe	8.04
**3a**	OMe	NH _2_	7.08
**3b**	OMe	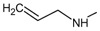	6.55
**3c**	OMe	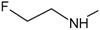	6.89
**3d**	OMe	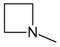	7.62
**3e**	OMe	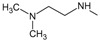	7.96
**3f**	OMe	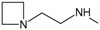	6.89
**3g**	OMe	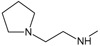	6.8
**3h**	OMe	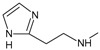	7.22
**15**	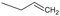	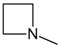	6.41
**a**	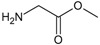	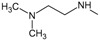	6.46
**4b**	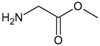	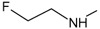	6.96
**4c**	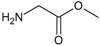	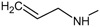	6.46
**4d**	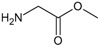	OMe	6.32
**4e**	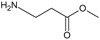	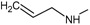	5.74
**4f**	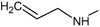	OMe	5.77
**6a**	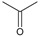	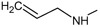	6.35
**6b**	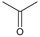	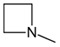	6.59
**6c**	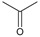	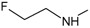	6.77
**6d**	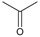	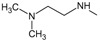	7.00
**7a**	H	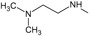	6.38
**7b**	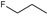	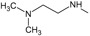	6.41
**7c**	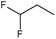	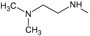	6.43
**7d**	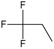	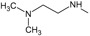	6.57
**7e**	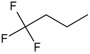	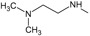	6.08
**7f**	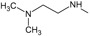	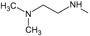	6.34
**8a**	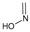	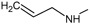	6.46
**8b**	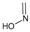	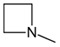	6.51
**8d**	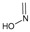	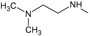	7.02
**8e**	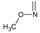	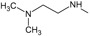	6.26
**8f**	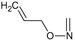	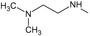	6.32
**8g**	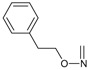	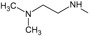	6.32
**8h**	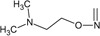	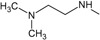	5.60
**8i**	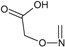	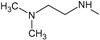	5.57

**Figure 2.**
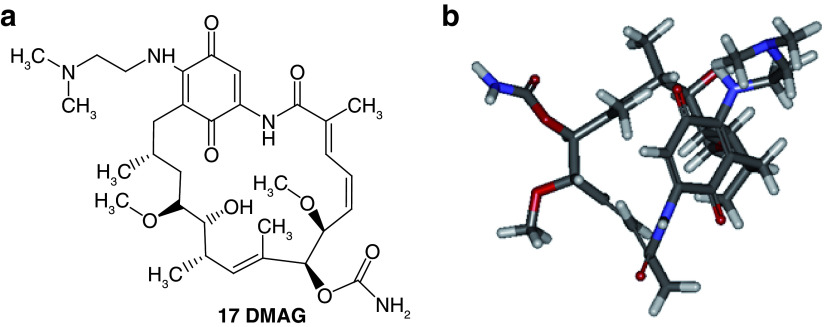
**a**) R ^3^ indicates the position that takes the substituents on the C-11 position of the macrocycle for the analogues of series 7 and **b**) Structure bioactive of the 17-DMAG.

***Design of structures***. For the design of the structures of the geldanamycin analogues, a template of the analog 17-DMAG from protein Data bank was obtained (code: 1OSF P; Figure 2). This template has a bioactive conformation ( Figure 2b), which was used to design the structures under study. For this, the substituents on C-11 and C17 of each analogue were incorporated using the design tool of Sybyl 7.3 (Tripos International) ^22^. Molden is an alternative software for design of structures, which can be downloaded for free.

**Figure 3.**
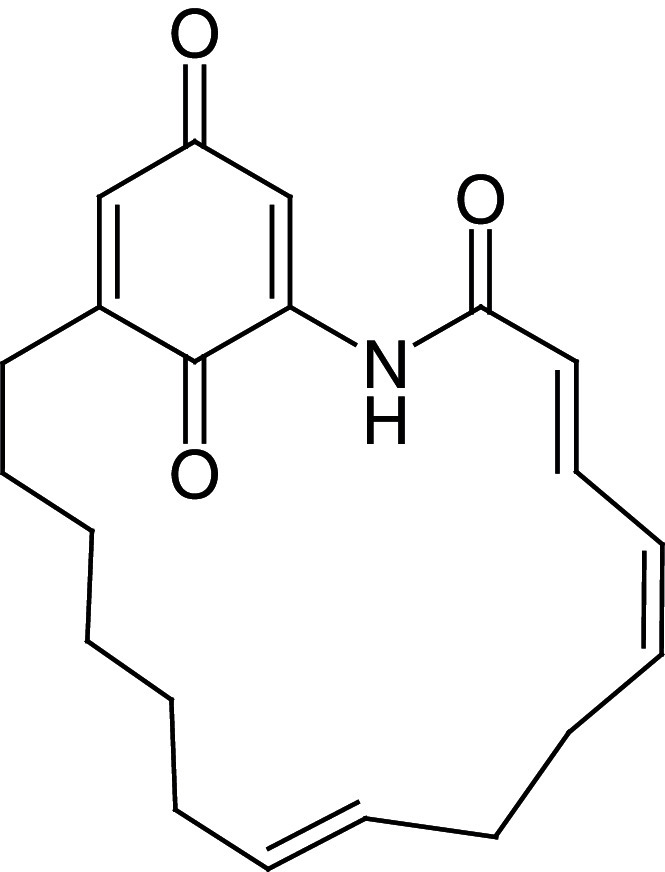
Structure of the geldanamycin macrocycle maintained as rigid during molecular dynamics.

***Optimization of structures***. Once the design of the structures for the geldanamycin analogues was completed, short molecular dynamics were performed. The macrocycle in all analogues were left rigid, as this did not lose its bioactive configuration ( Figure 3). The dynamics conditions were MM3 force field, 350 K, 5 ps of heating and 30ps of duration. Then, the conformations with lower energy were selected and carried out to partial optimization, using semi empirical approach PM6 method implemented to MOPAC 2009 ^23^.

***Docking***. The optimized structures of the geldanamycin analogues were taken to perform docking calculations. The process of docking was carried out for each of the analogues. Docking was implemented in the AutoDock program 1.5.4 version and its ADT 4.2 graphic interface ^24^. The parameters selected to perform the docking were completely flexible ligands, grid dimensions 50×50×50 Å with 0.375 Å spacing and generation of 100 poses for each ligand.

Once the results of the docking were obtained, the hydrogens were added to each of the poses obtained from AutoDock and then its optimization was carried out using PM6 semi-empirical method (AutoDock -PM6 hybrid methodology) implemented in MOPAC 2009. This procedure was carried out in order to recover the information represented in the interactions with other hydrogen atoms, which were not considered by AutoDock. This calculation also allowed information related to interaction energies at a more formal level of theory to be obtained. For the optimization with the semi-empiric method, amino acids residues of the active site within a radius of 5Å with center in the ligand was considered.

By the selection of the best poses of each methodology (AutoDock and AutoDock -PM6 hybrid methodology) the following parameters were considered: a) the pose reported by AutoDock with lower energy within the histogram of greater population and b) the best pose given by methodology hybrid (AutoDock-PM6) were selected according to the stability of complex (low energy).

Subsequently, three models of 3DQSAR were obtained from: a) simple alignment based on the superposition of structures optimized analogues of geldanamycin **(A model)**, b) alignment based on the superposition of structures obtained from docking ( **model B**), and c) alignment based on the superposition of structures obtained from the AutoDock PM6 hybrid methodology ( **model C**). Sybyl 7.3 program were used by alignments. The CoMFA approach was performed using PLS (Partial Least Squares) to a maximum of 10 components.

In contrast, the interaction energy of analogues-residue (I.E) was calculated to observe the behavior the geldanamycin analogues once these bound to the active site of HSP 90. Moreover, EI was used to explain the influence of interactions residues-analogues in the biological activities.

 For this, the complex between analogues and residues of active site (5 Å) of HSP90 were defragmented using Sybyl and PM6 semi-empirical method (AutoDock -PM6 hybrid methodology) combination. The interaction energy of analogues-residue (I.E) was calculated:

              Interaction energy of analogue -residue (I.E) = E _AM_ - E _L_-E _M_              

where E _AM_ a E _L_ and E _M_, denote analogue-residue energy, ligand energy and residue energy, respectively.

### Molecular quantum similarity measure: Generalities

The density functional theory (DFT) calculations were used to analyze the non-covalent stability of the compounds on the active site. TGSA algorithm was used to perform these calculations ^25a^. Quantum similarity and reactivity indices were used. A molecular quantum similarity measure (MQSM) between two systems A and B was performed. ZAB is a comparison between two molecules that can be constructed using their respective density functions (DFs). DFs can be multiplied and integrated over all the respective electronic coordinates, in turn weighed by a defined positive operator Ω(r _1_,r _2_) ^25b– 27^

$$Z_{AB}=\langle \rho_{A}|\Omega |\rho_{B}\text{〉}=\iint \rho_{A}\left(r_{1}\right)\Omega \left(r_{1},\;r_{2}\right)\rho_{B}\left(r_{2}\right)dr_{1}dr_{2}\;(1)$$

The nature of the operator used in Equation 1 will provide information that will be compared between the two systems and at the same time will name our measure of similarity; when the operator chosen is the Dirac delta function (a function that finds a very useful approach for functions with high peaks, such as electronic density, and constitutes the same type of mathematical abstraction such as the charge or the point mass). This is Ω(r _1_,r _2_)= δ(r _1_- r _2_), and we obtain an overlapping MQSM, one of the first similarity measures used; another widely handled possibility is the use of the Coulomb operator, that is Ω(r _1_,r _2_)= |r _1_- r _2_| ^-1^, obtaining a coulombic MQSM. A measure of similarity can be applied between two molecular systems, including the case in which the two molecules are equal, in this case the measurement is called measure of self-similarity (Z _AA_ for the case of molecule A) ^26^.

Given a group of N molecules, we can obtain, for each of them, a measure of similarity with respect to each of the group's molecules, including itself. From all these obtained measurements we can construct a symmetric matrix. The i-th column of the matrix can be considered as the collection of all measures of similarity between the i-th molecule and each element of the group, including itself. This is why each vector (each column of the matrix) can be considered as a discrete N-dimensional representation of the i-th structure. These collections of vectors can be considered as a group of molecular descriptors. However, this collection of columns of the similarity matrix does not simply constitute another group of molecular descriptors like those that are generally used to theoretically describe a given molecule; each descriptor has the following particular characteristics ^26, 27^:

**i**  Universality, in the sense that it can be obtained from any group of molecules and for any molecule within the group.
**ii**  Impartiality, since in the construction process there are no other options than those provided by the knowledge of the density functions and the similarity measures involved.

***Manipulation of MQSM and visualization techniques: Similarity indexes***. Once we have chosen a group of study objects and the operator related to the MQSM in Equation 1, the measure of similarity obtained for the group is unique; however, it is common practice to transform or combine these measures to obtain a new class of auxiliary terms that can be called quantum similarity indices (QSI). There is a vast amount of possible manipulations of MQSM that lead to a variety of QSI definitions. The most used are the following and by this reason are used in this work ^28– 30^:

Carbó's similarity index between two molecules I and J

$$C_{IJ}\left(\Omega \right)=z_{IJ}(\Omega ){\left[z_{II}(\Omega )z_{JJ}(\Omega )\right]}^{-1/2}\;(2)$$

This corresponds to the cosine of the angle subtended by the DFs involved, taken as vectors, for which this index is also called the cosine-like similarity index. This Carbo QSI, for any pair of molecules compared, it has a value between 0 and 1, which depends on the similarity associated with the two molecules (when I = J the index will approach 1) ^28– 30^.

Quantum similarity using Euclidean distance

Considering the similarity Equation 3,

$$D_{IJ}\left(k,x,\text{Ω}\right)=\;{\left[k\left(z_{II}\left(\text{Ω}\right)+z_{JJ}\;\left(\text{Ω}\right)\right)/2-xz_{IJ}\left(\text{Ω}\right)\right]}^{1/2},\;x\left[0,\;k\right]\;(3)$$

for which, if k = x = 2, it is reduced to the so-called Euclidean distance index. We can also define the index 3 of the form:

$$D_{IJ}\left(\infty ,\text{Ω}\right)=max\left(z_{II}\left(\text{Ω}\right),\;z_{II}\left(\text{Ω}\right)\right)\;(4)$$

This Equation 4 constitutes the distance index of infinite order ^31^.

***Molecular Quantum Similarity Measure (MQSM) definition used in this work***. The measures of quantum similarity are based on psychological perception and the obvious principle of similarity: “The more similar the two molecules are to one another, the more they are similar in their properties”. From this fact, we are able to obtain a quantitative measure of quantum similarity of the range of similarity between two molecules; they are based on the comparison of their densities. For the range of similarity between two compared systems. Generally, the MQSM is a measure between two tentative density functions involving molecular systems. The density functions are multiplied and integrated for the electronic coordinates in the convenient domain. MQSM between two systems A and B, denoted ZAB, is a comparison between two molecules that can be constructed using their respective Density Functions (DFs). Both DFs can be multiplied and integrated over all the respective electronic coordinates, in turn weighed by a defined positive operator Ω(r1,r2). TGSA software was used to carry out similarity measure ^25a^. The MSQM can be single defined at the scale of the first order molecular density functions associated with the compared molecules, and with positively defined operators ^26– 29^:

$$Z_{IJ}\text{Ω}=\text{〈}\rho_{I}\left|\text{Ω}\right|\rho_{J}\text{〉}=\iint \rho_{I}\left(r\right)\text{Ω}\left(r_{1},\;r_{2}\right)\rho_{J}\left(r_{2}\right)dr_{1}dr_{2}\in R^{\text{+}}\;(5)$$

where A and B are the two quantum objects studied, {r _1,_ r _2_} are the set of electronic coordinates associated with the corresponding wave function, {ρ _A_, ρ _B_} of the first order and Ω (r _1_, r _2_) positively defined supported in the operator, dependent on the coordinates of the electrons ^24– 29^.

***Types of measures in molecular quantum similarity***. It depends, essentially, on the information required, seriously on the selection of the supported operators, producing different types of MSQM. For these calculations the TGSA program was used ^25a^.

MQSM overlap considering Equation 2

The simplest and most intuitive usual choice of a positively defined operator is the distribution Dirac's delta, Ω (r _1_, r _2_) = δ (r _1_, r _2_). This selection transforms the general definition of MQSM, specifically, to calculate the overlap MQSM, which obtains measurements of the volume enclosed in the superposition of both electronic density functions ^26– 29^:

$$Z_{IJ}\text{Ω}=\iint \rho_{I}\left(r_{1}\right)\delta \left(r_{1}-\;r_{2}\right)\rho_{J}\left(r_{2}\right)dr_{1}dr_{2}=\int \rho_{I}\left(r\right)\rho_{J}\left(r\right)dr\;(6)$$

The Dirac delta function comes intuitively from physical definition and is computationally compliant. The MQSM comes from information on the concentration of electrons in the molecule and indicates the degree of overlap between the molecular comparison ^25b– 30^.

MQSM Coulomb considering Equation 2

If the operator (Ω) is adopted by the Coulomb operator, $\Omega \;\left(r_{1},\;r_{2}\right)=\frac{1}{\left|r_{1}-r_{2}\right|}$ , it provides the coulomb MQS, which represents the electrostatic repellent coulomb energy between two charge densities ^30, 31^:

$$Z_{IJ}\text{Ω}=\iint \rho_{I}\left(r_{1}\right)\frac{1}{r_{1}-\;r_{2}}\rho_{J}\left(r_{2}\right)dr_{1}dr_{2}\;(7)$$

The coulomb operator performs the effect for the overlap density functions. Considering the functions of molecular density as an electron distribution in space, this expression is only for the extension of Coulomb for the distribution of continuous charge, and for that reason it can be considered, in some occasions, as descriptors of electrostatic potential. This operator obtains the measurement of electrostatic repulsion between electronic distributions and is associated with electrostatic interactions ^25b– 29^.

Euclidean distance index considering Equation 3

This is another typical transformation that can be defined according to the classical distance:

dab=[∑j=1p(Δxj)k]1k(8)

where Δ *x _j_* = *x _aj_* – *x _bj_* is the distance between the objects a and b, and k=2 for the definition of distance. The Euclidean distance between two quantum objects A and B is defined by 25b– 29:

$$d_{ab}=\sqrt{{\left(x_{a}-x_{b}\right)}^{2}}.\;(9)$$

Occasionally it is expressed as: $D_{AB}=\sqrt{Z_{AA}+Z_{BB}+ZZ_{AB}}$ D _AB_ has values in the range of [0,∞﴿ but, converges for previous cases, it has a value of zero between the compared objects, if the compared objects are identical ^25b– 29^:

$$D_{AB}=0\;(10)$$

Geometrically this index can be interpreted by the norm of the differences between the density functions of the compared objects. The index of the Euclidean distance can be defined by the distance or dissimilarity index; the index can also be expressed as ^25b– 29^:

$$D_{AB}=\left\|\rho_{A}-\rho_{B}\right\|=\sqrt{{\left(\rho_{A}-\rho_{B}\right)}^{2}}\;(11)$$

***Alignment method: Topo-geometrical superposition algorithm (TGSA)***. In this work, the alignment was carried out using the TGSA ^32^ method. The TGSA was proposed by Gironés and programmed and implemented by the same author. This method considers that the optimal alignment of molecules is carried out through superposition on the common skeleton, taking only into account the type of atoms and the bond of the interatomic interactions, which is the atomic number of the coordination. Was carry out, its purpose the algorithm examines the atomic pairs of the molecules and aligns the common substructure for a series of molecules ^32^. The method is only based on topology and geometric considerations, where the molecular topology is manifested in the way of comparing the distant bonds. In two molecules, the superposition is unique and does not depend on the type of operator chosen to provide the meaning of the similarity ^32^.

First, molecular coordination and atomic number are necessary to indicate the performance of the program. The molecular coordination is ordered in bases, according to the decrease of the atomic number, in order to determine a path for the number of hydrogens in the molecule ^32^.

Considering that the superposition of hydrogens is not significant, and with the required computational requirements, the hydrogen atoms are not included in the process. The next step is the definition of the atomic pair, the duo is defined only if the pair of atoms in the box is involved, with their respective determinants, the duo has to be defined for each molecule, all molecules behave with each other with their respective meanings of interatomic distances, obtaining translocations. The translocations are taken within the fluctuations of the spine of the conformations produced by the presence of the different substitutions in the molecules ^32^. This procedure always discards bonds that are not common with skeletons. Once the duos are compared, the algorithm creates atomic triads by adding three atoms selected from the duos. These supplementary atoms must be in the box to be compared later. In geometric terms, this generates a triangle in the plane, where the atoms occupy the vertices of the triangle, and the sides correspond to the effectiveness of the chemical box ^32^.

The triangle obtained by a molecule is compared with the triangle obtained by the second molecule with the respective interatomic distances, and with the translational distances in the comparison duo. If the three distances of both triangles compared are similar, both triads are similarly considered and stored. The triads that do not meet the classification criteria are automatically discarded to complete the comparison, the selected triad is superimposed and the result of the molecular alignment is determined univocally ^32^.

This process is repeated for the atoms and the algorithms chosen are those of the alignment that maximizes the number of atoms superimposed, minimizing the index C _IJ_, it is used by the comparison criterion of the interatomic distances and this cost calculation with the absolute value of each difference with the composite after-location:

$$C_{IJ}:\sqrt{\frac{\sqrt{d_{II}d_{JJ}}}{d_{IJ}}}\;(12)$$

where $d_{IJ}:\sum_{i:1}^{n_{A}}\sum_{j:1}^{n_{B}}{\left|x_{i,I}-x_{j,J}\right|}^{2}$ , n is the number of atoms and x the molecular coordination, C _IJ_ is determined in the interval [0,1], evaluating the quantification of the overlap. This indicates better the alignment when C _IJ_ it approaches unity, originating the ideal case of structural identity C _IJ_: 1.

The TGSA method considers the molecules as rigid bodies, so there is no flexibility in the structure (nothing of rotation and vibration in the distances of the angles in the box). This is designated by the operator in the homogeneous set of molecules and does not yield good results with different molecular structures; this comes from the alignment pair that is restricted with the common recognition skeleton. In contrast, this common recognition of substructures produces a coherent alignment with chemical intuition. TGSA-Flex program was used to perform this procedure because is simple and has low computational requirements ^32– 36^.

### DFT based reactivity descriptors

Some of the present authors have shown the relationship between quantum similarity and chemical reactivity descriptors in several works ^37– 45^. In addition, the quantum similarity and DFT use the DF as an object of study (B3LYP/631G*). The similarity indexes, specifically the Coulomb index, can be related to electronic factors associated with chemical reactivity. All the calculations were carried out using the free software TGSA-Flex ^32^

Using the Frontier Molecular Orbitals (FMO) and the energy gap, the global reactivity indices, such as chemical potential (μ) ^45– 47^, hardness (η) ^45– 47^ and electrophilicity (ω) ^45– 47^, will be calculated. These chemical reactivity indices give an idea about the stability of the systems.

The chemical potential (μ) characterizes the tendency of the electrons to escape from the equilibrium system, whereas the chemical hardness (η) is a measure of the resistance of a chemical species to change its electronic configuration ^46, 47^.

$$\mu \approx \frac{E_{LUMO}+E_{HOMO}}{2}\;(13)$$

and

$$\eta \approx E_{LUMO}-E_{HOMO}\;(14)$$

Electrophilicity index can be interpreted as a measure of the stabilization energy of the system when it is saturated by electrons from the external environment and is mathematically defined as ^39– 43^:

$$\omega \approx \frac{\mu^{2}}{2\eta }\;(15)$$

In this work, the local reactivity descriptor are the Fukui functions ( Equation 16 and Equation 17, *f*). The Equation (16) and Equation (17) represents the response of the chemical potential of a system to changes in the external potential. It is defined as the derivative of the electronic density with respect to the number of electrons at constant external potential:

$$f_{k}^{+}\approx \int_{k}\left[\rho_{N+1}\left(\vec{r}\right)-\rho_{N}\left(\vec{r}\right)\right]=\left[q_{k}\left(N+1\right)-q_{k}\left(N\right)\right]\;(16)$$

$$f_{k}^{-}\approx \int_{k}\left[\rho_{N}\left(\vec{r}\right)-\rho_{N-1}\left(\vec{r}\right)\right]=\left[q_{k}\left(N\right)-q_{k}\left(N-1\right)\right]\;(17)$$

where ( $f_{k}^{+}$ ) is for nucleophilic attack and ( $f_{k}^{-}$ ) for electrophilic attack ^44– 46^. In this sense, using the global and local reactivity descriptors it is possible to study the quantum dissimilarity along the molecular set.

## Results and discussion

### Statistical results of the 3DQSAR models

Table 2 shows the results obtained for the prediction descriptors (q ^2^) and correlation (R ^2^) of the three models.

From the three models that were evaluated, only C model provided the best prediction values (q ^2^) to all components evaluated. However, the change in q ^2^ to 2, 5 and 7 components (48.3, 46.8 and 49.0 %, respectively) was not significant, which infers that C model was more reproducible than A and B models, as observed in Table 2.

**Table 2.** Statistical prediction descriptors (q ^2^) and correlation (R ^2^) of the three models, 3D QSAR to 2, 5 and 7 components.

	3D QSAR (A Model)			3D QSAR AUTODOCK (B Model)			3D QSAR AUTODOCK-PM6 (C Model)		
**NO. COMPONENTS**	2	5	7	2	5	7	2	5	7
**q ^2^**	0.411	0.194	0.183	0.366	0.425	0.336	0.483	0.468	0.490
**R ^2^**	0.607	0.815	0.871	0.698	0.943	0.984	0.672	0.929	0.963

**Figure 4.**
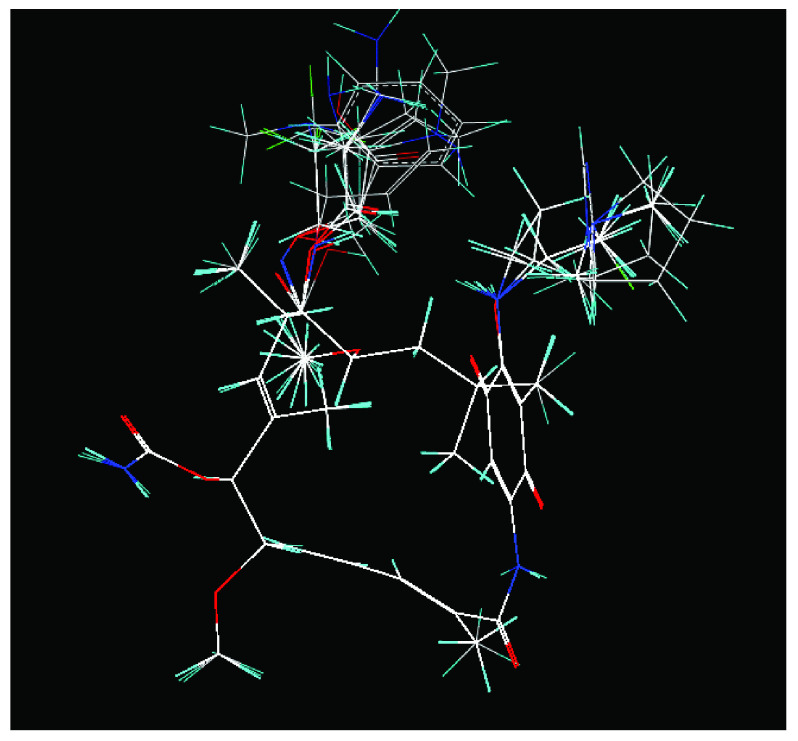
Alignment used to obtain 3DQSAR model A.

**Figure 5.**
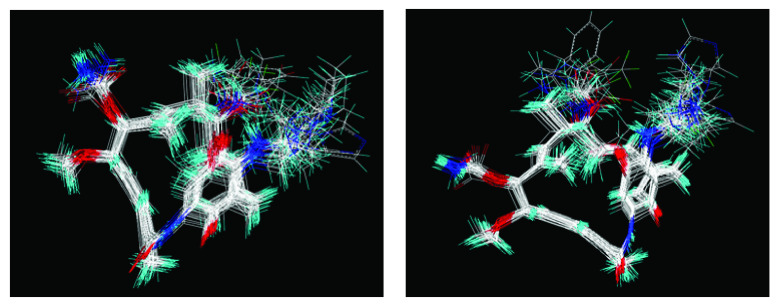
Alignment used for models 3DQSAR B and C, respectively.

In addition, the results obtained from B and C models provide information about receptor ligand-interactions, which can be observed in the alignments of these models ( Figure 4 and Figure 5); where the conformational changes observed in analogues structures can be evidence of what may be happening at the cellular level. However, the C model is superior to B model because all hydrogens atoms were considered in receptor-ligand coupling (complex), while, B model only polar hydrogen atoms. Thus, model C is more real in terms of the interactions of analogues with the active site of HSP90 (Quantum mechanical calculations), thus providing a new alternative for docking studies ^7, 48^. The results obtained to A model were lowest; and this fact can be associated to alignment used to generate 3DQSAR, where the structures used by alignment were only optimized without considered the interaction with active site. Thus, the only difference was observed in the aligned structures were on C-11 and C-17; which are positions where geldanamycin analogues are structural different ( Table 2).

### Electrostatic and steric maps derived from CoMFA

**Figure 6.**
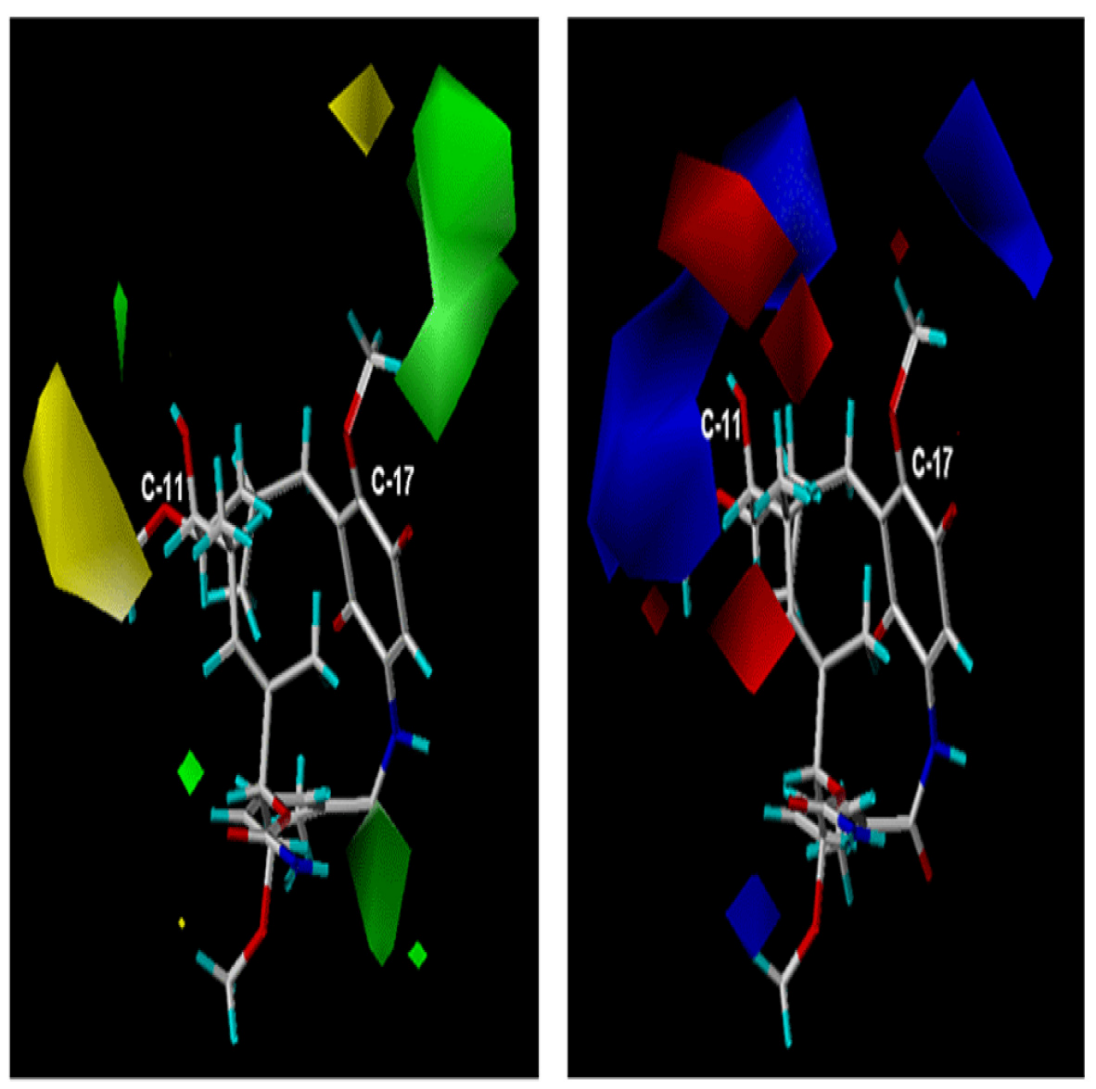
( **a**) Steric map and ( **b**) electrostatic map on positions C-11 and C-17 (CoMFA).

The model **C** (CoMFA) shows the contribution of the steric and electrostatic fields which generated R ^2^= 0.963 and q ^2^= 0.50 for an optimal number of 7 components. On the other hand, the steric and electrostatic individual contributions were 57.6 and 42.4%, respectively. Steric and electrostatic contour maps are shown in Figure 6.

In Figure 6a and 6b, steric and electrostatic contours are depicted. These contours are located in a special way on positions C-11 and C-17 macrocycle, this is expected because these are the positions where the structural differences of the geldanamycin analogues occur.

The steric CoMFA map ( Figure 6a) covers the sterically favorable contours (80% contribution) corresponding to the regions in the space where the steric volume leads to an increase in activity (green contour map), while the sterically non favorable regions (20% contribution) correspond to areas in the space where the steric volume is expected a decrease of activity. Map of green steric contour on the carbon atom C-17 position of the macrocycle indicates analogues with bulky substituents in this position tending to favor the activity, as it is reflected in the 3e ligands (pIC _50_ = 7.96) and 1f (pIC _50_ = 7.77) that have this peculiarity on carbon 11. The yellow outline indicates that the presence of bulky groups adversely, affected activity, as noted in the 4f ligands (pIC _50_ = 5.77) and 8i (pIC _50_ = 5.57). This analysis of steric maps allows us to infer that the ligands of series **1** and **3** are favored, while to the ligands of series **8** – **7** ( Table 1 and Table 2) are disadvantaged.

On the electrostatic map ( Figure 6b), the blue contours (80% contribution) indicate the regions in the space where the groups with low electron density favor activity, while the red contours (20% contribution) indicate regions where groups with high electron density decrease activity.

The blue contours on the C-17 position of the macrocycle indicate that the activity is favored for those ligands with substituent electroatractors in that position (ligands 1e pIC _50_ = 7. 62 and 3 h pIC50 = 7. 22). In contrast, the red and blue contours on the C-11 position of the macrocycle indicate that both the electronegative and electropositive substituents can promote activity provided that they should be oriented towards the contours on the map. This counts the vast majority of the ligands in this study that can present any of the two conditions set out for the substituents of C-11. General examples of this behavior are the ligands **1g** (pIC _50_ = 7. 15) and **3d** (pIC _50_ = 7. 62) oriented towards the blue contour. **4b** (pIC _50_ = 6. 99), **3a** (pIC _50_ = 7. 08), **3b** (pIC _50_ = 6. 55) are oriented towards the red contours. It can be seen that the steric map (CoMFA) is the best explanation of the relation between structure-activity of the geldanamycin analogues, since the contours obtained from those analogues are more specific than the electrostatic ones, and give a clearer indication of the type of substituents that should be on positions C-11 and C-17 of the macrocycle.

To confirm the outcomes of this, we carried out other calculations in order to explain the behavior of this set of molecules.

**Figure 7.**
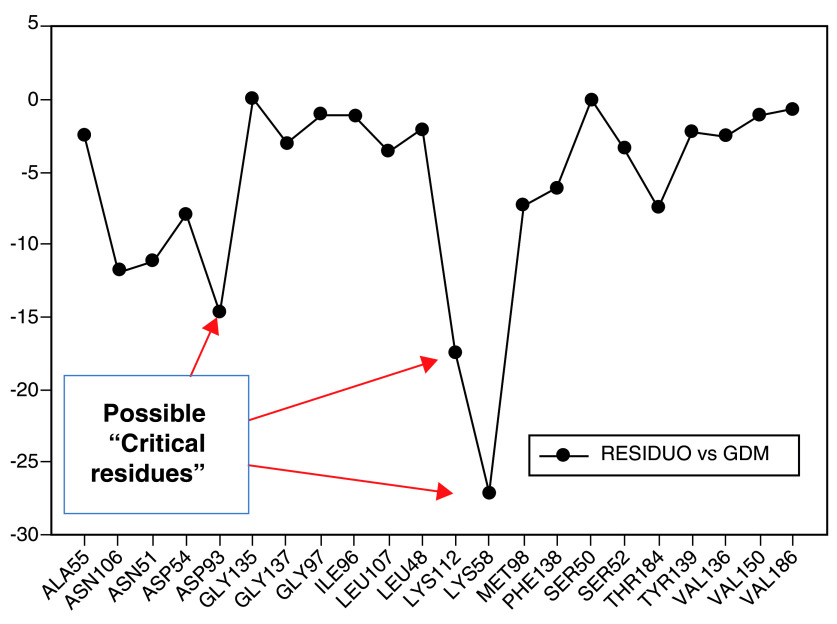
Interaction energies of geldanamycin. Red arrows show the energy interaction values of Lys58, Asp93 and Lys112 residues.

While the results of the 3DQSAR were clear, they did not fulfill expectations, because they cannot explain, in all cases, the behavior between structure-activity. For this reason, the energy of interaction (I.E) residue-ligand was calculated. In order to observe the activity within the active site of Hsp90, once this was bonded to the geldanamycin analogues an accurate analysis of the interactions ligand (substituents)-receptor could be obtained. In Figure 7, the interaction energies obtained for geldanamycin have been plotted.

In Figure 7, it is shown that the most significant values of I.E are present in Lys58, Asp93, Lys112 residues, where Lys58 is the most favorable of them, which suggests that this could possibly be a "critical" residue to analyze. On the other hand, it is expected that the interaction with the residue Lys58 is highly favorable because of the ease that has this residue forms a bridge of hydrogen with oxygen of the hydroxyl of the C-11 position of the macrocycle group.

**Figure 8.**
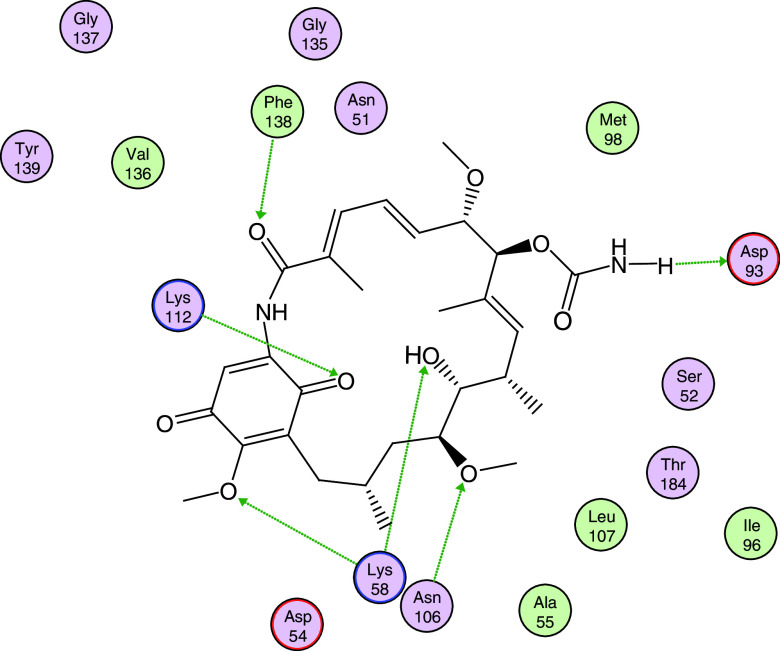
Possible interactions between residues of the active site of Hsp90 and geldanamycin. Green lines indicate the residues donator of hydrogen bridge.

In Figure 8, the interactions between geldanamycin and the active site of Hsp90 is depicted, and it can be observed that the possible "critical" residues can interact positively with geldanamycin, hence why the I.E may have been significant. Moreover, Lys58 can donate a bridge of hydrogen with methoxy group (C-17) and hydroxy group (C-11), but the interaction is the most favored for hydroxyl group, because the methoxy group is far from the active site ^26^. The above does not rule out that the C-17 methoxy group does not affect the energy of the interaction with this residue.

**Figure 9.**
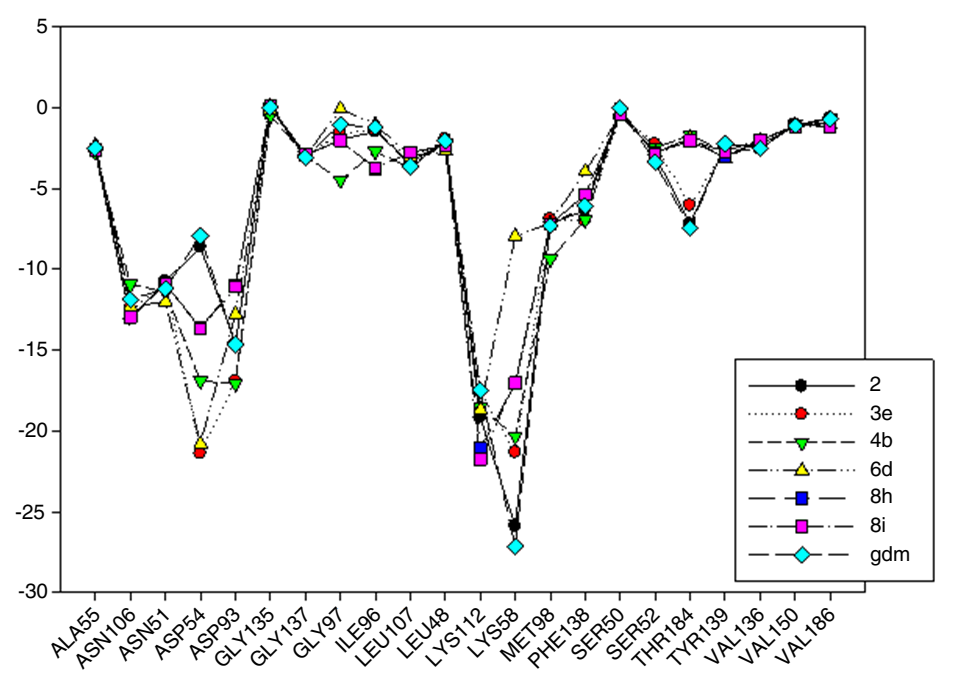
Interaction energies of geldanamycin analogues 2, 3e, 4b, 6 d, 8 h, 8i and geldanamycin.

In Figure 9, interaction energies for the composite 2 (pIC _50_= 8.04) and 3e (pIC _50_= 7.96) have been plotted. 4b (pIC _50_ = 6.96), 6d (pIC _50_= 7), 8i (pIC _50_= 5.6) and 8h (pIC _50_= 5.57) present high, medium and low activities, respectively, like their predecessor.

The choice of these analogues was made in order to show the changes that have the values of I.E possible “critical" residue when making cellular modifications on C-11 and C-17. This could corroborate the outcomes obtained which are similar to geldanamycin and Lys58, Asp93 and Lys112 residues. In addition, the significant values of I.E with Asp54, which can be considered as another possible "critical" residue. Interaction with Asp54 is justified by the high electron density that this residue has, which allows it to act as an acceptor of the hydrogen bridge of the substituents on C-17, which are potential donors, for instance, the substituents possessing an amine or diamine on that position ( Table 1 and Table 2)

In Figure 9, it was also noted that Lys58 and Asp54 present a significant gap in values of I.E with respect to the Lys112 and the Asp93, which in turn means that interactions with these geldanamycin analogue residues are of high relevance. Interestingly, it was also found that those residues are located on positions C-11 and C-17 of the macrocycle, where precisely the substitutions occur (see Figure 8). To verify the importance of residues Lys58, Asp93, Lys112 and Asp54 as "critical” residues, it was necessary to examine the analogues of geldanamycin by family, because the structural differences are minimal.

**Figure 10.**
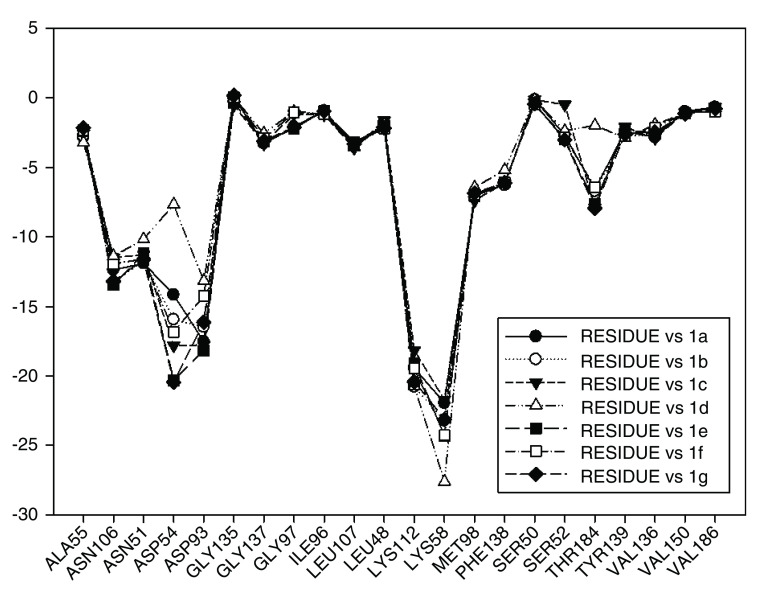
Interaction energies of 11-hydroxy analogues and geldanamycin.

**Figure 11.**
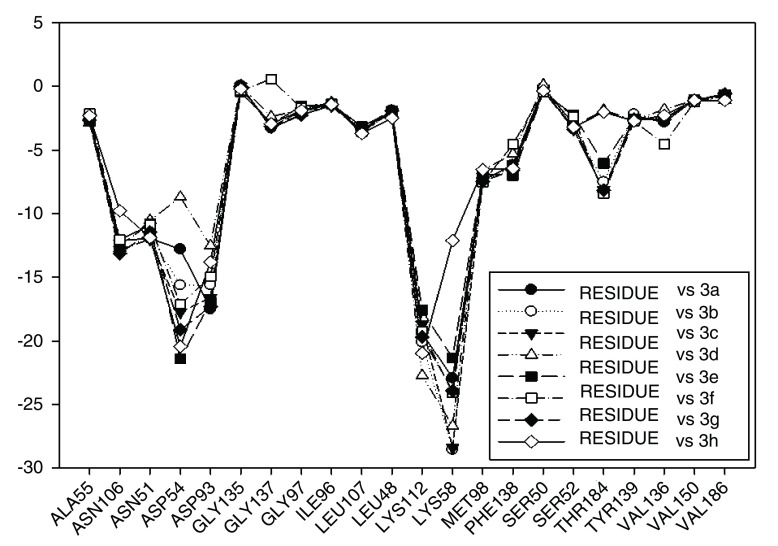
Interaction energies of 11-methoxy geldanamycin analogues.

Continuing with the analysis of interaction energy, we considered series 11-hydroxy and 11-methoxy together because these only differ in substituent on C-11. In Figure 10 and Figure 11, the values of I.E for 11-hydroxy and 11-methoxy of the geldanamycin analogues have been plotted. In these figures, it can be observed that most of the series of compounds tend to have significant I.E values with Lys58, Asp93 and Lys112 and Asp54 residues, which makes us suppose that those residues are "critical" for both series.

The highest values of I.E were found in Lys58 and the Asp54 ( Figure 10 and Figure 11). The I.E for the Lys58 values tend to be higher and homogenous for 11-hydroxy than for 11-methoxy series, since the Lys58 forms a hydrogen bridge with oxygen more easily (the macrocycle position C-11). Besides, this group does not have major steric or repulsive impediments. In the case of 11-methoxy series, the interaction is disadvantaged because of the steric impediment that presents with the methyl group. But despite this, in both series the atom of oxygen, by effects of polarization of bond, holds a partial negative charge, which in turn makes it a good bridge acceptor of hydrogen (effect observed in all analogues of both series). However, the I.E. values for Asp54 are more fluctuating for both series since some analogues of these series cannot donate a hydrogen bridge to the residue, as is the case of the analogues 1d and 3d that lack an atom of hydrogen in the substituent at C-17 ( Table 1). Hence, the low values of I.E with this residue (-7.6577 and -8.70502, respectively), which in turn leads to interactions with Gly 137, Gly97, Ile96, and Met98 residues (1b analogue). The above may be occurring because the analogues of 11-methoxy series tend to rearrange within the active site in a way that can interact with Lys58, which in turn implies it will strengthen interaction with other residues.

This behavior allows to the assumption that interaction between Lys58 and substituent on C-11 are important for biological activities of analogues with hydroxy and methoxy groups on C-11. Moreover, these analogues have the same substituent on C-17 and thus the same interaction with Asp54.

**Figure 12.**
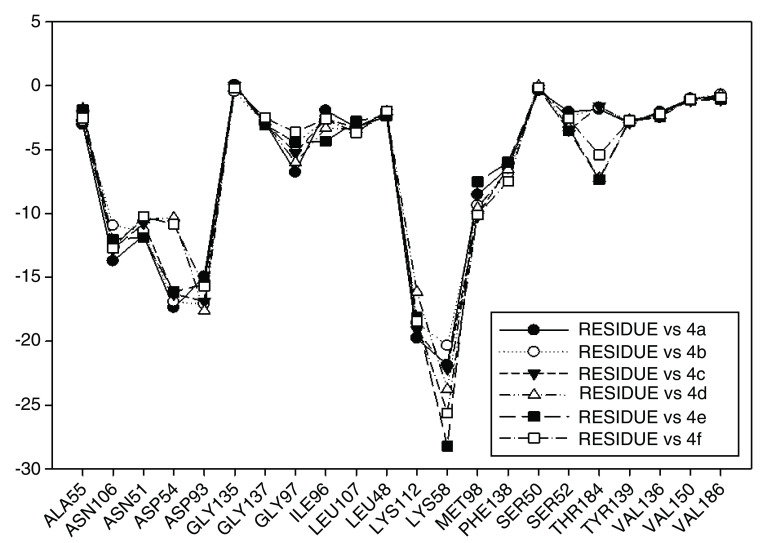
Interaction energies of 11-O-acyl geldanamycin analogues.

In Figure 12 the values of I.E for 11-O-acyl geldanamycin analogues have been plotted, which show the trend seen in the two previous series, since most of these compounds loses the interactions with Gly97 and Ile96 residues, and the values of I.E for Asp54 residue are similar to the Asp93. However, the trend of significant values of I.E for Lys58 as observed in previous cases is the same. The behavior observed in this series can be associated to the steric impediment of the substituents on C-11 and C-17, which impedes the formation of hydrogen bridges with Lys58 and Asp54 residues. On the other hand, as explained above, the steric impediment in these molecules induced a change, which involves the regrouping of these residues on the active site, and thus, these have to stabilize through the formation of new interactions with other residues such as Gly97 and Ile96. Also in Figure 12, it can be observed that for analogues of this series, despite having bulky groups in C-11, the interaction with Lys58 remains the highest, which suggests that the interaction with this residue has a high relevance to these analogues, and that to increase the interaction decreases the interaction with Asp54. It is of note that the analogues that cannot make a bridge of hydrogen to Asp54 have a low I.E value, as was observed with 4e and 4d analogues, which possess a methoxy group on C-17. Moreover, the non-formation of a hydrogen bond creates repulsion between oxygen atom and carboxyl group of this residue. In general, this series highlights that Lys58 acts as a "critical" residue and that to some extent it can affect the activity of these compounds ^7, 49^.

**Figure 13.**
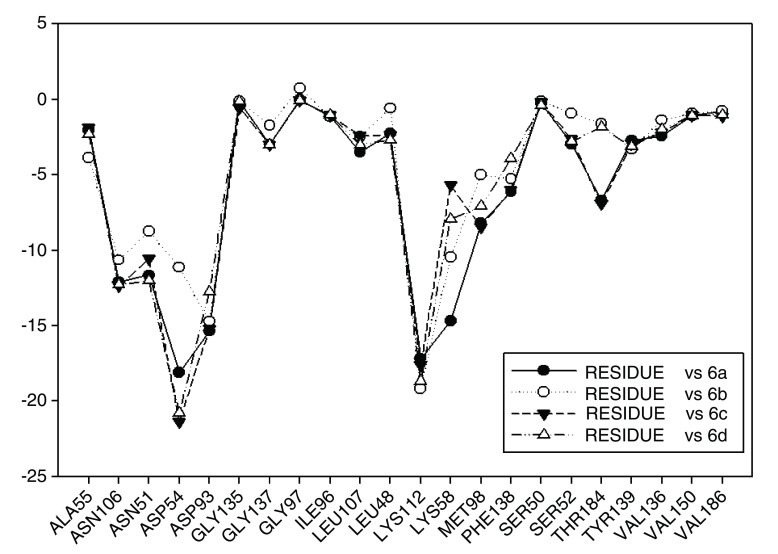
Interaction energies of 11-ketone geldanamycin analogues.

In Figure 13 the values of I.E for 11-ketone analogues of geldanamycin have been plotted. It can be observed that the I.E values with Lys58 are not significant as in the previous cases because Lys112, which happens to be the residue with the best I.E value, displaces these. This behavior can be explained due to the carbonyl content, which easily accepts the bridge of hydrogen donated by the Lys58 as would be expected. The preferences that have these analogues by the Lys112 residue can occur because of the short distance between carbonyl group and this residue. Besides, the rigidity that presents the double bond of the carbonyl group on C11 shields hydrogen formation with Lys58 and thus the molecule suffers a rearrangement within the active site, leading it to seek other interactions enabling it to stabilize. This was observed with Met98, Phe138 and Lys112, which had the best iteration energy.

Figure 13 also shows the values of I.E for the Asp54 residue. The behavior observed was similar to that observed with the other series, but increases the interaction with Asp54 that made the interaction with Lys58 decrease. In general, with analogues of this series it can be deduced that the formation of a weak interaction with Lys58 forces the molecule to strengthen its interaction with other residues of the active site, in order to stabilize. As was previously mentioned these groups of compounds differ from other geldanamycin analogues since these only have substitution in the hydrogen of the amine located in C11 ( Figure 2a) since the substituent at C-17 is the same for all analogues of this series (see Table 2).

**Figure 14.**
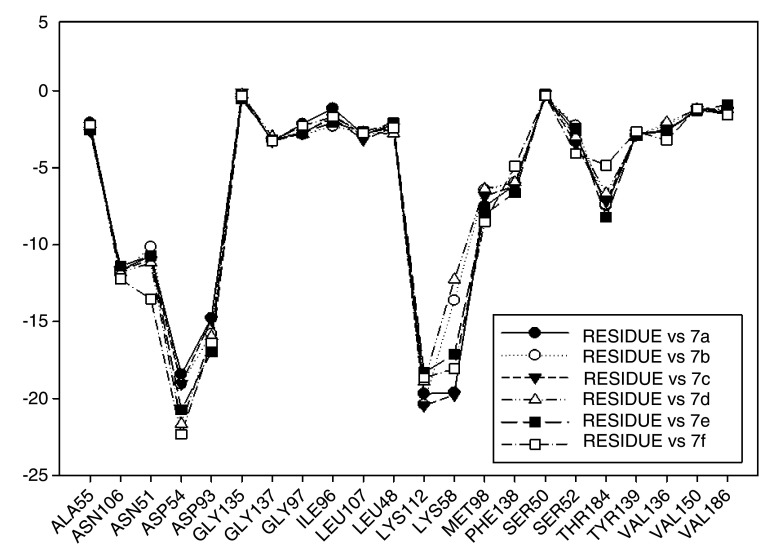
Interaction energies of 11-amine geldanamycin analogues.

**Figure 15.**
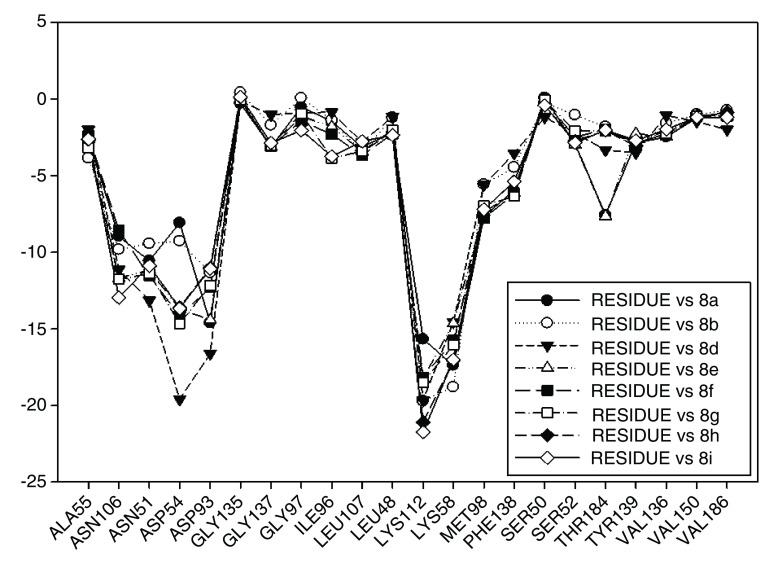
Interaction energies of 11-oxime geldanamycin analogues.

Figure 14 plots the values of I.E for 11-amine analogues and Lys112, which were more significant than those found for Lys58. This is expected because the substituents on C-11 are not the most propitious to create interaction with Lys58, because these do not possess an atom which can accept a hydrogen bond. Moreover, the volume of these substituents ( Table 2), induce a reorganization in the interactions with the active site, as was observed with Lys112, Asp93, Thr184 and Asp54 residues. The latter has special mention because, as seen in Figure 15, its values of I.E are the highest, which in turn allows us to infer that in the absence of a good interaction with Lys58, the compounds in this series are stabilized in particular to strengthen its interaction with Asp54 and subsequent to this with Lys112, Asp93 and Thr184. Further interactions with Asp54 are more favored, since the substituent on C-17, can donate a hydrogen bond to the residue, when the substituent is voluminous.

In general, the fall of values of I.E for Lys58 could be due to the low activity of these compounds, which is complemented by the considerable volume of the substituents on C-11. On the other hand, the interaction these analogues have with Asp54 serves to give stability to the complexes, especially for those analogues of geldanamycin possessing a secondary amine on C-17. Figure 15 also plots the values of I.E for 11-oxime analogues of geldanamycin.

The I.E values for Lys112 are higher than Lys58 ( Figure 15), with the exception of 8a where its behavior is opposite (Lys58: -17,3721 vs Lys112: -15,6787). This could be due the fact that 8a has an atom of oxygen on C-11, which can accept, without any steric problems, a hydrogen bridge with Lys58. In addition, the substituent on C-17 is the least bulky of this series, which favors their interaction. 8b and 8d possess the same substituent on C-11, so would be expected to have the same values for I.E with Lys58 as 8a (-18.8069 and -14.561, respectively), and thus these were higher than with Lys112 (-19.6651 and -18.2331, respectively). However, this behavior is not observed for the similar substituents on C-17, where these induce a change that involves strengthening the interactions with other residues to compensate the decrease of interaction with Lys58. This behavior was particularly observed in the interactions with Lys112 where this was higher than Lys58.

For the rest of the compounds of this series (8e-8i), the I.E values are to be expected to be low for Lys58 because the substituents at C-11 are voluminous analogues ( Table 1), which prevents the formation of the hydrogen bridge by steric effects with Lys58, since the molecule has to be rearranged within the active site in such a way that this interaction is what gives it a certain degree of destabilization to the molecules and gives the force to strengthen interactions with other residues (Lys112). The above is supplemented by the fact that the substituents at C-17 are also bulky.

In general, the interaction between substituents on C-11 and Lys58 could have an impact on geldanamycin analog activity. However, the interaction between C-17 and Asp54 also could have an important role in activity, since substituent volume on C-11 can affect C-17 because this molecule gives priority to certain points in its site active. 

**Figure 16.**
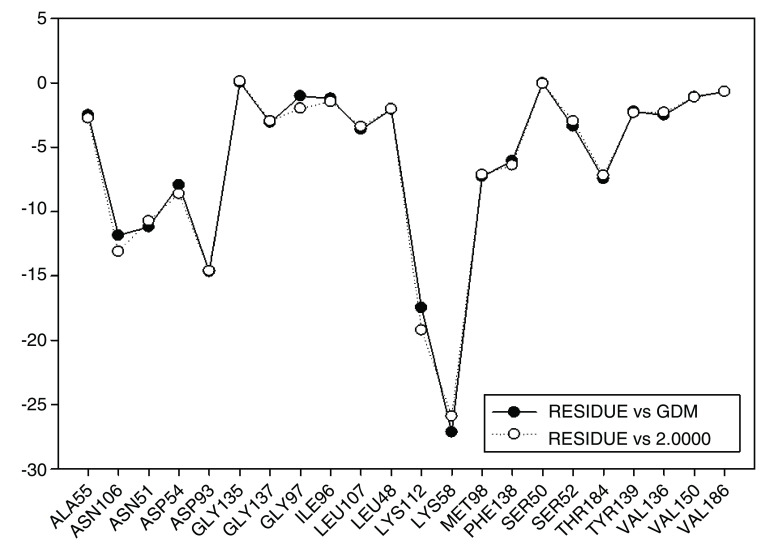
Comparison of the interaction energies of geldanamycin and analog 2.

These results are compared to I.E value for geldanamycin and the most active analogue 2 ( Figure 16). It can be observed that I.E value for residues in both compounds do not have significant differences, which may explain the increase in the activity of 7.39 (GDM) to 8.04 (2). Thus, it can be expected that the activities of these compounds are similar or failing that, the activity of compound 2 is slightly superior to the GDM. The above tells us it would be worth reviewing the value of the biological activity of this ligand.

### Molecular quantum similarity indices to the most reactive compounds with reference compound GMD and reactivity analysis: Analysis of the non-covalent interactions on the active site

To understand the 3D-CoMFA outcomes, a quantum similarity study on the most reactive compounds was performed. Taking into account the reference compound GDM, in Table 3 are shown the overlap similarity indices using Equation 12. These indices allow us to make some considerations about the quality of the superposition on the molecular set. The compound with the higher structural similarity with the reference compound GDM is **1a** (0,9837, Table 3) with a Euclidean distance of 1,0662 (see Table 5). Therefore, the group (-NH _2_) in compound **1a** shows a light dissimilarity in the alignment method. This result agrees with the low steric effect to this group.

**Table 3.** Molecular quantum similarity indices using the overlap operator in Equation 12.

O_Hab	1a	1b	1c	1d	1e	1f	2	3d	3e	GDM
**1a**	**1,0000**									
**1b**	0,9171	**1,0000**								
**1c**	0,8881	0,9097	**1,0000**							
**1d**	0,9128	0,9338	0,9234	**1,0000**						
**1e**	0,8924	0,9122	0,9689	0,9280	**1,0000**					
**1f**	0,8875	0,9658	0,8792	0,9041	0,8876	**1,0000**				
**2**	0,9584	0,9215	0,9019	0,9516	0,9044	0,8942	**1,0000**			
**3d**	0,57168	0,5840	0,5680	0,5588	0,5295	0,5682	0,6025	**1,0000**		
**3e**	0,5177	0,5868	0,5720	0,5293	0,5234	0,5874	0,5714	0,9411	**1,0000**	
**GDM**	0,9837	0,9244	0,8799	0,9262	0,8892	0,8962	0,9631	0,6675	0,6489	**1,0000**

The compound with lowest similarity with respect to GDM is compound **3e** (0,6489, see Table 3) with a Euclidean distance of 5,1217 (see Table 4). Compound **3e** has two substituents groups with high steric effects according to Table 1. The bulky substituents can show some problems on the superposition process. Compound **2** has higher biology activity **(**pIC _50_=8.04, see Table 1) and has an overlap index with respect to the reference compound 0,9631 with an Euclidean distance of 1,6289. These compounds have two methoxy groups that are electron-donating groups. These electronic considerations have strong influence in the superposition process due to the fact that flexibility and free movement on ramifications decrease.

**Table 4.** Euclidean distance using the overlap operator in Equation 11.

D_O_Hab	1a	1b	1c	1d	1e	1f	2	3d	3e	GDM
**1a**	**0,0000**									
**1b**	2,4288	**0,0000**								
**1c**	2,8848	2,6148	**0,0000**							
**1d**	2,4908	2,1946	2,4093	**0,0000**						
**1e**	2,8208	2,5704	1,5509	2,3277	**0,0000**					
**1f**	2,8973	1,6199	3,0678	2,6971	2,9526	**0,0000**				
**2**	1,7269	2,3930	2,7255	1,8791	2,6837	2,8339	**0,0000**			
**3d**	5,53943	5,5302	5,7313	5,6964	5,9673	5,7366	5,4120	**0,0000**		
**3e**	5,9742	5,5979	5,7874	5,9753	6,0943	5,6886	5,7064	2,1310	**0,0000**	
**GDM**	1,0662	2,3266	2,9949	2,2990	2,8695	2,7911	1,6289	4,9025	5,1217	**0,0000**

**Table 5.** Molecular quantum similarity indices using the Coulomb operator in Equation 12.

C_Hab	1a	1b	1c	1d	1e	1f	2	3e	3d	GDM
**1a**	**1,0000**									
**1b**	0,9897	**1,0000**								
**1c**	0,9877	0,9957	**1,0000**							
**1d**	0,9917	0,9941	0,9940	**1,0000**						
**1e**	0,9791	0,9919	0,9962	0,9895	**1,0000**					
**1f**	0,9746	0,9927	0,9880	0,9831	0,9893	**1,0000**				
**2**	0,9969	0,9914	0,9899	0,9953	0,9829	0,9779	**1,0000**			
**3e**	0.9658	0.9694	0.9663	0.9710	0.9473	0.9599	0.9710	**1,0000**		
**3d**	0.9553	0.9691	0.9689	0.9626	0.9545	0.97164	0.9597	0.9888	**1,0000**	
**GDM**	0.9994	0.9904	0.9869	0.9914	0.9794	0.9754	0.9968	0.97397	0.96363	**1,0000**

The main intention of this study was to observe the electronic effects of the group substitutes and Coulomb indices. Table 4 shows the values obtained with the most reactive compounds with respect to the reference compound GMD. Table 5 depicts the electronic similarity indices using the Coulomb similarity to analyze the electronic effects on group substitutes.

Owing to inductive effects, the basicity of the amine group might be expected to increase with the number of alkyl groups on the amine. However, correlations are complicated owing to the effects of solvation, which are opposite to the trends for inductive effects, according to what is shown in the 3D-CoMFA maps. However, solvation effects also dominate the basicity of aromatic amines. For these compounds, the lone pair of electrons on nitrogen delocalizes into the ring, resulting in decreased basicity. Substituents on the aromatic ring, and their positions relative to the amine group also affect basicity, as seen in Table 1. To understand these electronic features, Table 6 shows the global chemical reactivity descriptors to the most reactive compounds.

**Table 6.** Euclidean distance using the overlap operator in Equation 11.

D_C_Hab	1a	1b	1c	1d	1e	1f	2	3d	3e	GDM
**1a**	**0,0000**									
**1b**	11,3734	**0,0000**								
**1c**	12,6133	7,1659	**0,0000**							
**1d**	10,4700	8,2672	8,3949	**0,0000**						
**1e**	17,1556	10,4691	7,3056	11,7298	**0,0000**					
**1f**	18,9751	10,2728	12,6568	14,9220	11,7298	**0,0000**				
**2**	6,6216	9,9815	10,9230	7,4543	14,9219	17,0594	**0,0000**			
**3e**	2,0450	1,91621	2,0137	1,8656	2,5608	2,2547	18634	**0,0000**		
**3d**	2,44730	2,0085	2,0094	2,19810	2,4249	1,92103	2,2831	1,2256	**0,0000**	
**GDM**	2,4112	11,0023	2,9243	10,6019	7,4543	18,6799	6,6793	1,8058	2,2377	**0,0000**

The compound with higher global reactivity descriptors is 1c with chemical potential (-4,9587 eV), hardness (5,6360 eV), softness (0,1774 eV) ^-1^ and electrophilicity (2,1814 eV). The reference compound GMD has chemical potential (-4,8982 eV), hardness (5,5895 eV), softness (0,1789 eV) ^1^ and electrophilicity (2,1462 eV). The compound with higher biology activity 2 has chemical potential (-4,5953 eV), hardness (5,2243 eV), softness (0,1914 eV) ^-1^ and electrophilicity (2,0210 eV) (see, Table 7). The high hardness and electrophilicity values in these compounds may be related with the non-covalent stabilization on the active site. Electrophilicity is related with the system saturation when received electrons come from the external environment. To analyses these reactivity details, the Frontier molecule is shown in Figure 16– Figure 18, and show orbitals HOMO, LUMO and the Fukui Functions $\text{〈}f_{k}^{-}\text{〉}\approx {\left|HOMO\right|}^{2}$ and $\text{〈}f_{k}^{+}\text{〉}\approx {\left|LUMO\right|}^{2}$ , to compounds 1a, GMD (reference compound) and 2.

**Table 7.** Global reactivity descriptors to the most reactive compounds.

Compound	C. Potential (eV)	C. Hardness (eV)	Softness (eV) ^-1^	Electrophilicity (eV)
**1a**	-4,6761	5,4932	0,1820	1,9903
**1b**	-4,6608	5,4494	0,1835	1,9931
**1c**	-4,9587	5,6360	0,1774	2,1814
**1d**	-4,5300	5,0529	0,1979	2,0306
**1e**	-4,5975	5,2009	0,1923	2,0320
**1f**	-4,5529	5,3625	0,1864	1,9327
**2**	-4,5953	5,2243	0,1914	2,0210
**3d**	-4,5229	5,0523	0,1979	2,0245
**3e**	-4,5296	5,3177	0,1880	1,9292
**GDM**	-4,8982	5,5895	0,1789	2,1462

**Figure 17.**
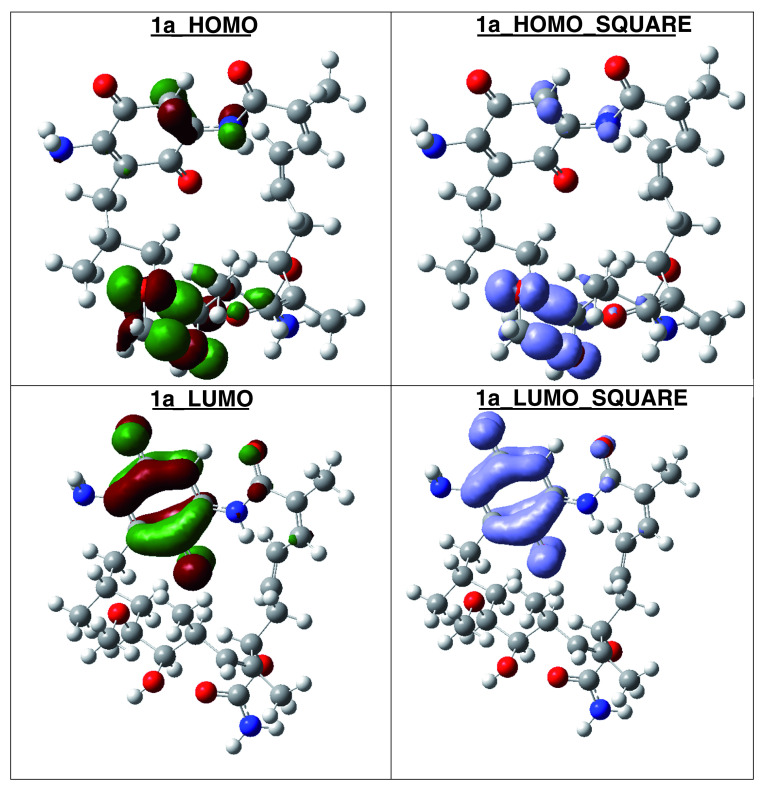
Frontier molecular orbitals HOMO, LUMO (isosurface 0.02), the Fukui Functions $\text{〈}f_{k}^{-}\text{〉}\approx {\left|HOMO\right|}^{2}$ and $\text{〈}f_{k}^{+}\text{〉}\approx {\left|LUMO\right|}^{2}$ (isosurface 0.004), to compound 1a.

Compound **1a** has the higher structural and electronic similarity with **GDM** (reference compound) and compound **2** has the higher biological activity.

**Figure 18.**
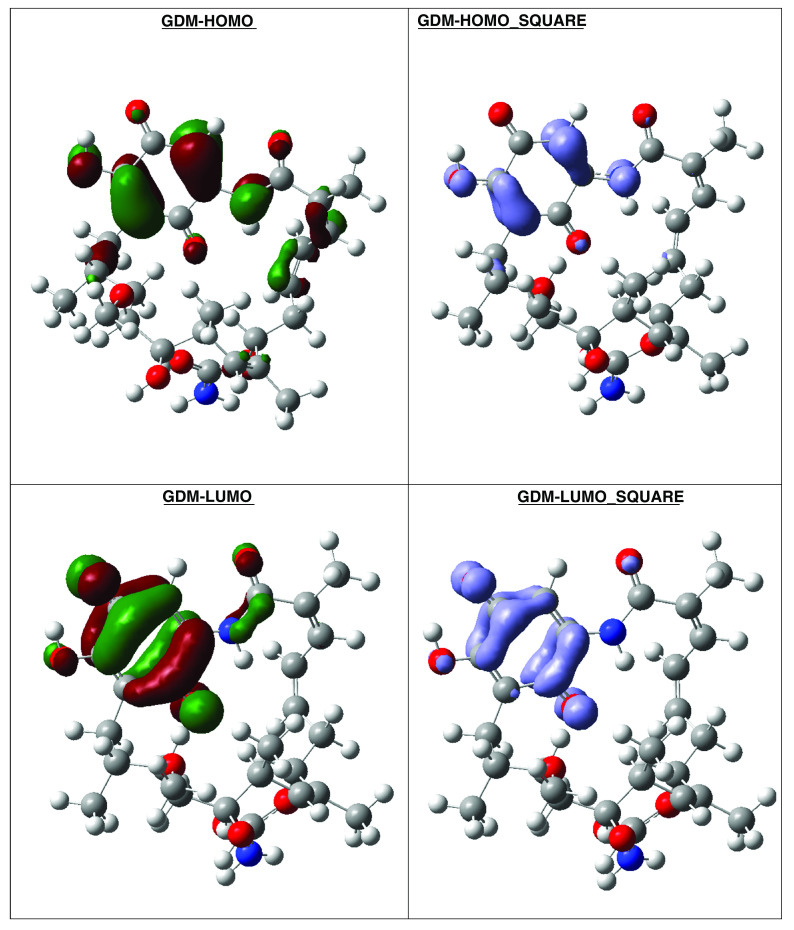
Frontier molecular orbitals HOMO, LUMO (isosurface 0.02), the Fukui Functions $\text{〈}f_{k}^{-}\text{〉}\approx {\left|HOMO\right|}^{2}$ and $\text{〈}f_{k}^{+}\text{〉}\approx {\left|LUMO\right|}^{2}$ (isosurface 0.004), to reference compound GDM.

**Figure 19.**
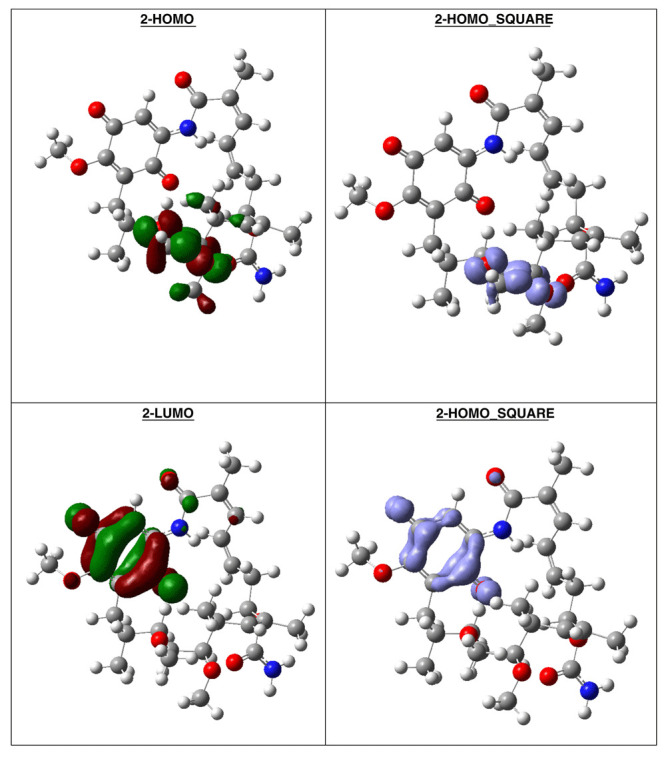
Frontier molecular orbitals HOMO, LUMO (isosurface 0.02), the Fukui Functions $\text{〈}f_{k}^{-}\text{〉}\approx {\left|HOMO\right|}^{2}$ and $\text{〈}f_{k}^{+}\text{〉}\approx {\left|LUMO\right|}^{2}$ (isosurface 0.004), to reference compound 2.

Compound GDM show that the Fukui Functions are in the same zone, and these reactivity characteristics are very important, since these can be related with nucleophilic character on the active site and non-covalent stabilization (see Figure 19).

The reactivity contours in compound 2 are very similar to compound 1a. Therefore, these compounds have different regions to electrophilic and nucleophilic attacks. Therefore, we can see the different effects that can occur in the central ring when different substituents such as activators or deactivators are used.

## Conclusion

The application of Autodock-PM6 hybrid methodology allowed to select the best interactions between residues of Hsp90´s site active and geldanamycin analogues, which, allowed to improve the results of 3D QSAR. With the data provided by the energy of interaction, we suggest that interactions that influence the activity of the geldanamycin analogues are those formed with residues such as Lys58, Lys112 and Asp54, which we refer to as "critical" residues. This is supported by the fact that the substituents are bulky at position C-11 and the macrocycle does not favor the interaction with Lys58, and the substituents with amines secondary in C-17 from the macrocycle position favor the interaction with Asp54.

Subsequently the energy profile of each group of geldanamycin analogues showed that more active compounds have high energy of interaction with Lys58 and are less active with Lys112, regardless of the substituents onC-17. Consequently, the increase in I.E of Lys112 or Asp54 generates increased interaction energy with other residues and decrease the values of I.E with Lys58

According to our results, this research contributes with new methodology that can be used to improve the prediction from 3DQAR model, considering that the alignment employed for model generation is derived of the amino acid residues- compound interactions (hybrid methodology -Autodock-PM6).

## Data availability

### Underlying data

Figshare: Receptor_Alim_2019_Pose_E_D_1.mdb (1).zip, https://doi.org/10.6084/m9.figshare.10255739.v1 ^50^.

Data are available under the terms of the Creative Commons Attribution 4.0 International license (CC-BY 4.0).
