# Supplementary material for: Study of interaction energies between residues of the active site of Hsp90 and geldanamycin analogues using quantum mechanics/molecular mechanics methods
Source: F1000Res. 2020 Apr 16;8:2040. Originally published 2019 Dec 2. [Version 2] doi: 10.12688/f1000research.20844.2 (PMC10521063; doi:10.12688/f1000research.20844.2)
Supplement: Peer review history

# Reviewer report: Reviewer response for version 1

**DOI:** 10.5256/f1000research.22924.r58209

**Published:** 2020-02-20

**Authors:** Leonardo C. Pacheco-Londoño

**Affiliations:** 1 R3-C Research and Education Component of ALERT DHS Center of Excellence for Explosives Research, Department of Chemistry, University of Puerto Rico, Mayaguez, PR, USA; 2 School of Basic and Biomedical Science, Universidad Simón Bolívar, Barranquilla, Colombia

The authors of "Study of interaction energies between residues of the active site of Hsp90 and geldanamycin analogues using quantum mechanics/molecular mechanics methods" describe the implementation of the methodology QSAR to explain the biological activity of geldanamycin analogues. Overall, the design of the calculus is good, and the results are of reasonable quality. There are several points that the authors should address before indexing in F1000Research:

1. The authors must clearly explain the contribution to the state of the art of this study. Apparently, the QSAR methodology doesn’t have new information, if there is something new in it, this should be highlighted in the summary and conclusion. It is possible that the novelty of the study is the explanation of how the geldanamycin analogues interact with Hsp90. Please emphasize this.
2. The authors generate models with 43 of 48 analogues that were reported in reference 21. The other 5 are not used because they do not report their IC _50_ value. Why is the activity of these 5 not predicted?
3. Figures 7, 9 11-16: the y-axes have no units (EI?).
4. Apparently, the authors do not indicate the method of DFT used for the calculations of reactivity descriptors.
5. The direct application of this study is the use of models to predict or propose new molecules with more activity. As a recommendation, the authors should propose other molecules of this type that present greater activity. It would be more interesting to study for those who are working on the synthesis of these molecules.

Is the work clearly and accurately presented and does it cite the current literature?

Yes

If applicable, is the statistical analysis and its interpretation appropriate?

Yes

Are all the source data underlying the results available to ensure full reproducibility?

Yes

Is the study design appropriate and is the work technically sound?

Yes

Are the conclusions drawn adequately supported by the results?

Yes

Are sufficient details of methods and analysis provided to allow replication by others?

Yes

Reviewer Expertise:

My area of expertise related to the article is theoretical calculations to explain points that through experimentation are not possible to explain or complementing experimental data. The area in which I have worked the most is the design of sensors using vibrational spectroscopy, other areas where I unwind are the design of materials for biosensors and environmental applications

I confirm that I have read this submission and believe that I have an appropriate level of expertise to confirm that it is of an acceptable scientific standard.

# Response

**Published:** 2020-03-31

**Authors:** Ricardo Vivas-Reyes

**Affiliations:** Universidad de Cartagena, Colombia

**1. **The authors must clearly explain the contribution to the state of the art of this study. Apparently, the QSAR methodology doesn’t have new information, if there is something new in it, this should be highlighted in the summary and conclusion. It is possible that the novelty of the study is the explanation of how the geldanamycin analogues interact with Hsp90. Please emphasize this.

***Answer:*** *Thanks for your comments. The information was included in the manuscript.*

**

**2. **The authors generate models with 43 of 48 analogues that were reported in reference 21. The other 5 are not used because they do not report their IC50 value. Why is the activity of these 5 not predicted?- **5**. The direct application of this study is the use of models to predict or propose new molecules with more activity. As a recommendation, the authors should propose other molecules of this type that present greater activity. It would be more interesting to study for those who are working on the synthesis of these molecules.

***Answer:*** *Thanks for your comment. The information was not included because the objective of the study was to know that amino acid residues were key to the biological activity of the compounds and that groups of substituents on C-11 and C-17 improved the activity. The prediction of the activities is intended for a second article, where new analogs are also proposed.*

**3.** Figures 7, 9 11-16: the y-axes have no units (EI?).

***Answer:*** *Thanks for your comments. The information was included in the manuscript.*

**

**4. **Apparently, the authors do not indicate the method of DFT used for the calculations of reactivity descriptors.

***Answer: **Was done.*

# Reviewer report: Reviewer response for version 1

**DOI:** 10.5256/f1000research.22924.r58072

**Published:** 2020-01-30

**Authors:** Jaime Enrique Plazas Román

**Affiliations:** 1 Corporación Universitaria Rafael Núñez, Cartagena, Colombia

The importance of this article lies in the active site of Hsp90 which is involved in the process of cellular oncogenesis; Geldanamycin is currently being used, however due to the hepatotoxic damage showed in clinical trials by Geldanamycin, it is necessary to look for Geldanamycin analogs. This article finds or gives solutions to this question by giving as a result through docking, 3DQSAR and quantum similarity of these analogs, so it is an interesting and outstanding result on clinical research, excellent work.

 I think this paper is excellent and is an important addition to the literature. The evaluation of interaction energies from the interaction with Lys58 residue is essential for the union of the analogues to the active site of Hsp90, and improves its biological activity. This union is formed through a substituent on C-11 of the geldanamycin macrocycle. A small and attractor group was found as the main steric and electrostatic characteristic that substituents on C11 need in order to interact with Lys 58; behavior was observed with hydroxy and methoxy series of geldanamycin analogues, under study.

Is the work clearly and accurately presented and does it cite the current literature?

Yes

If applicable, is the statistical analysis and its interpretation appropriate?

Yes

Are all the source data underlying the results available to ensure full reproducibility?

Yes

Is the study design appropriate and is the work technically sound?

Yes

Are the conclusions drawn adequately supported by the results?

Yes

Are sufficient details of methods and analysis provided to allow replication by others?

Yes

Reviewer Expertise:

bioinformatics, biological computation

I confirm that I have read this submission and believe that I have an appropriate level of expertise to confirm that it is of an acceptable scientific standard.

# Response

**Published:** 2020-03-31

**Authors:** Ricardo Vivas-Reyes

**Affiliations:** Universidad de Cartagena, Colombia

Thank you very much for your comments
